# Long term safety, tolerability, and efficacy of intracutaneous zolmitriptan (M207) in the acute treatment of migraine

**DOI:** 10.1186/s10194-021-01249-z

**Published:** 2021-05-17

**Authors:** Stephanie J. Nahas, Nada Hindiyeh, Deborah I. Friedman, Nada Elbuluk, Donald J. Kellerman, Pamela K. Foreman, Peter Schmidt

**Affiliations:** 1 Department of Neurology, Jefferson Headache Center, Thomas Jefferson University, Philadelphia, PA USA; 2 Department of Neurology, Stanford University Medical Center, Stanford, CA USA; 3 University of Texas Southwestern Medical Center, Dallas, TX USA; 4 University of Southern California, Keck School of Medicine, Los Angeles, CA USA; 5 Zosano Pharmaceuticals, Fremont, CA USA

**Keywords:** Migraine, M207, Microneedle, Intracutaneous

## Abstract

**Objective:**

To determine the long-term safety and tolerability profile of M207 in the acute treatment of migraine.

**Background:**

M207 is an investigational microneedle-based system for intracutaneous delivery of zolmitriptan for the treatment of migraine attacks. Following on the positive results of a Phase 2/3 placebo-controlled efficacy study (ZOTRIP), this study was designed to evaluate the safety of this novel product during repeated use for the treatment of migraine attacks.

**Methods:**

In this 6–12 month open-label, multicenter observational study, participants used an eDiary to record headache symptoms and adverse events at specified intervals up to 48 h following treatment of a qualifying attack with M207 3.8 mg (intracutaneous zolmitriptan). Participants underwent clinical evaluations at specified intervals up to 12 months.

**Results:**

Among 335 participants who treated ≥1 migraine attack, 257 completed 6 months and 127 completed 1 year of treatment. The most common reason for withdrawal from the study was a low frequency of reported attacks post randomization. Overall, 5963 migraine attacks were treated. Most participants (96%) experienced at least 1 adverse event, the vast majority of which concerned the application site, and > 95% of which were mild. Fifteen participants (4%) withdrew due to adverse events; 4 withdrew due to 7 application site reactions, 6 of which were mild.

Participants achieved pain freedom in 2477/5617 (44%) of attacks, most bothersome symptom freedom in 3315/5330 (62%) of attacks, and pain relief 2 h post-dose in 4552/5617 (81%) of attacks. Sustained pain freedom 2–24 h was seen in 1761/4698 (38%) of attacks, and 2–48 h in 1534/4429 (35%) of attacks.

**Conclusions:**

The majority of participants experienced cutaneous adverse reactions such as application site erythema, swelling, and bleeding, and most reactions were scored as mild. These results are consistent with what was observed in the single migraine attack treatment ZOTRIP trial indicating that M207 is well tolerated in the setting of longer-term repeated use. Efficacy findings were also similar to those in the ZOTRIP trial.

**Trial registration:**

Clinicaltrials.gov on September 13, 2017 (NCT03282227).

## Background

**Fig. 1:**
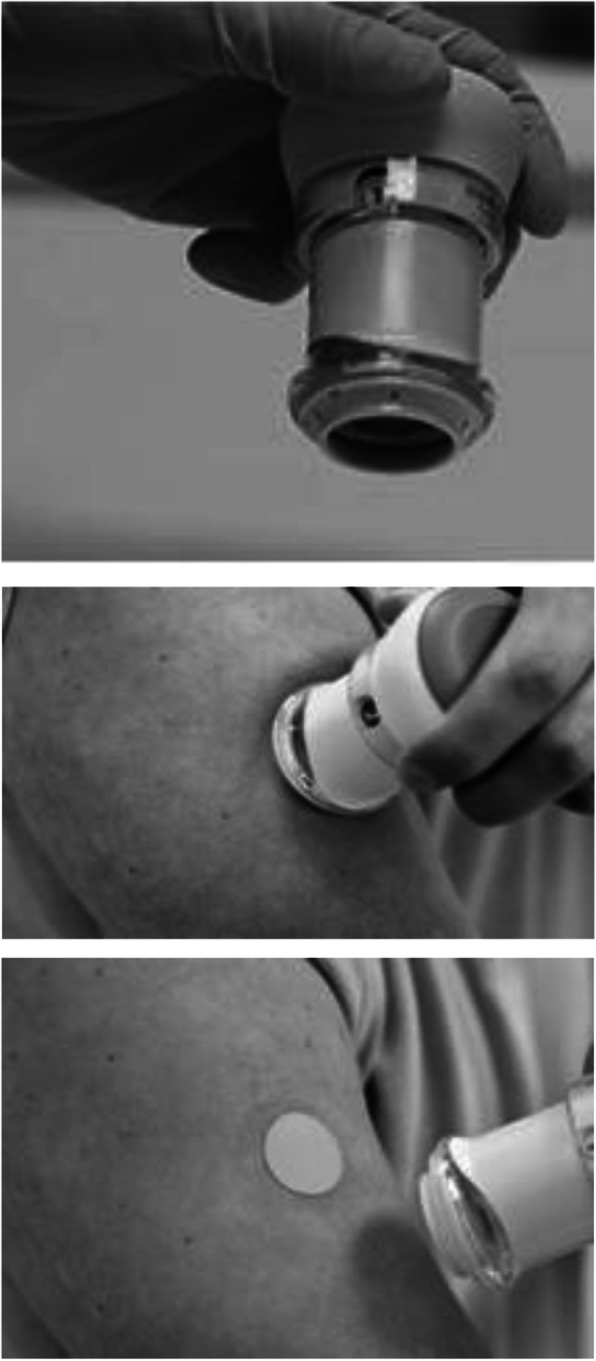
M207 system for zolmitriptan delivery. Table adapted from Reference [1], published under CC BY-NC-ND license

M207 is an investigational microneedle-based system for intracutaneous delivery of zolmitriptan for the treatment of migraine attacks (Fig. 1). In a phase 1 trial, M207 provided faster absorption (T_max_ of 15–20 min) with a greater 2-h exposure than oral zolmitriptan [1]. In a Phase 2b/3, double-blind, placebo-controlled trial (*N* = 365) in which M207 3.8 mg was used to treat a single migraine attack (ZOTRIP, NCT02745392), 2-h pain freedom was achieved by 41.5% of those on treatment compared to 14.3% of those on placebo [2]. Two-hour freedom from most bothersome symptom (MBS, i.e., photophobia, phonophobia, or nausea) was achieved by 68.4% of those on treatment compared with 42.9% of those on placebo [2]. M207 was well-tolerated in this trial, with 51.8% of M207-treated and 18.1% of placebo participants experiencing treatment emergent adverse events (TEAEs), the vast majority of which were considered mild [2]. A considerable percentage reported application site redness (26.5% in the M207 group and 10.8% in the placebo group) and bruising (14.5% M207 and 3.6% placebo); both were mild and transient in most participants.

Although microneedling systems are commonly used in dermatology, this was the first *combination* drug-device therapeutic microneedle system to reach Phase 3 of clinical development. Because the product is intended for repeated use in the outpatient setting, a long-term safety study was undertaken to assess the tolerability of M207 when used to treat migraine attacks repeatedly over a six- to twelve-month period. To assess application site reactions in real-time in the at-home setting, we utilized an eDiary to prompt self-assessments after every patch application. Participants were seen periodically by investigators, who examined and systematically scored patch application sites, and were queried about any non-skin-related adverse events they experienced.

This combination of solicited, participant-reported, real-time assessment of application site observations, investigator-assessments, and conventional adverse event assessment was incorporated as a rigorous design for evaluating the safety and tolerability of this new, unique drug delivery system. We report here the methodology and key findings from this study that support a well-characterized safety profile.

## Methods

This was a 6–12 month open-label, multicenter study conducted in the United States to assess the long-term safety and efficacy of M207 in the treatment of migraine attacks (NCT03282227). The primary outcome measure was the percentage of participants experiencing treatment emergent adverse events (TEAEs) over 12 months. Secondary outcomes included the percentage of migraine attacks for which pain freedom and MBS freedom were achieved at 2 h post-dose, and the percentage for which pain relief was achieved at 2 h post-dose.

The protocol was approved by the Copernicus (Cary, NC) and individual investigator site Institutional Review Boards. Potential participants provided written informed consent. Participants 18–75 years of age were eligible if they had a ≥ 1-year history of episodic migraine (according to ICHD-3 criteria) with onset prior to 50 years of age. Per history, participants must have experienced between 2 and 8 migraine days per month during the 6-month period prior to screening and may have had no more than 15 headache days per month.

The study consisted of a screening period, a run-in period of 2–3 weeks to determine eligibility, and a treatment period of up to 12 months. Following consent and screening, participants were issued an eDiary to record attack symptoms and adverse events (AEs), including skin reactions. Enrollees were eligible for treatment if they experienced at least 1 qualifying migraine attack and no more than 7 headache days during the run-in period. A qualifying migraine attack must have had at least two of the following: unilateral location, pulsating quality, moderate or severe pain intensity, interference with routine physical activity. In addition, the headache must have been accompanied by nausea or vomiting, or both photophobia and phonophobia.

During the treatment period, M207 was applied to the upper arm to self-administer a single dose of 3.8 mg of zolmitriptan as two 1.9-mg patches to treat each qualifying migraine attack. The second patch was applied next to the first one, and both were worn simultaneously for 30 min. Participants were instructed to use the eDiary to record migraine symptoms and application site observations pre-dose, and 30 min, 2 h, 12 h, 24 h, and 48 h post-dose. They were prompted by their eDiary to report whether bleeding was present (at 30 min only), and if erythema, edema, or bruising were present at the time of patch removal (30 min), and 2, 12, 24, and 48 h post-dose. Questions about pain or itching at the application site were asked at 48 h post-dose. A total of 18 questions were asked with responses recorded after each treated attack. Participants received a reference card to help them grade the application site findings on a 0–3 scale using images from preclinical studies. Any self-reported scores above 0 were reported as adverse events.

Migraine prophylaxis medications were allowed if participants had taken a stable dose for at least 30 days prior to screening and with no changes during the study. Aspirin, acetaminophen, nonsteroidal anti-inflammatory drugs, and other medications taken specifically for migraine symptoms were prohibited on the day of a confirmed migraine attack prior to M207 application and for 2 h after patch application.

Participants underwent clinical evaluation, including skin assessments, at months 1, 2, 3, 6, 9, and 12 of the treatment period, and a safety evaluation 2 weeks after discontinuation. Erythema, edema, and bruising were assessed by the investigator using the rating scale of none, mild (present over ≤25% of the application area), moderate (present over ≥26% to ≤50% of the application area), and severe (present over > 50% of the application area). Enrollees were queried about AEs and new concomitant medication use.

Efficacy assessments at 30 min, 2, 12, 24, and 48 h post-dose included the proportion of attacks with pain freedom, the proportion with freedom from a participant’s specified most bothersome migraine-associated symptom, and the proportion with pain relief. The primary timepoint of interest for efficacy was 2 h post-dose. Sustained pain freedom for 2–24 h and 2–48 h was also determined.

### Statistics

Descriptive statistics were used to assess demographics, AEs (from verbal reports as well as eDiary), and headache symptom responses. Missing data were not imputed; percentages displayed are out of the number of patients or attacks with data. To ensure adequate exposure to M207 to evaluate safety, the predefined goal for enrollment was a sufficient number of participants such that at least 150 participants treated an average of at least 2 attacks per month for 6 months, and at least 50 treated an average of at least 2 attacks per month for 12 months. All statistical tabulations and analyses were done using SAS® Version 9.4 or higher.

## Results

A total of 490 individuals were screened for participation in the study, and 342 were enrolled. Among them, 335 treated at least one migraine attack (safety population). Two hundred fifty-seven participants completed 6 months in the trial, and 127 participants completed 1 year of treatment. The most common reasons for not completing the trial were not having an average of 2 qualifying migraine attacks per month (*n* = 79) and sponsor’s decision to end the trial once participation goals had been achieved (*n* = 60). Fifteen participants (4.5%) withdrew from the study due to an AE.

The demographics of the study population were similar to many reported migraine trials. The mean age was 42.9 years, 88.7% of subjects were women, 79% were Caucasian, and 16% were Black or African American.

At baseline, photophobia was prespecified as the MBS in 51.9%, phonophobia in 23.2%, and nausea in 24.8%.

The average (standard deviation) number of treatments per month for all participants was 1.8 (0.91). Related to pre-defined participation goals, 162 participants treated on average at least 2 migraine attacks per month for 6 months, and 89 treated on average at least 2 migraines per month for 12 months. A total of 96 participants treated at least 25 migraine attacks and had post-treatment assessments. Overall, 5945 migraine attacks were treated and had at least one post-treatment assessment. In total, participants were prompted by the eDiary approximately 98,000 times to record their application site observations. Missing post-dose data rates for eDiary responses were as follows: 4.1% at 30 min, 5.5% at 2 h, 11.6% at 12 h, 8.7% at 24 h, and 10.1% at 48 h.

The AEs occurring in ≥2% of participants are shown in Table 1. The most common adverse events were related to the application site, and all were recorded in the participants’ eDiaries except for one instance of bruising and one instance of application site pain. Investigators’ assessments in the clinic revealed a similar pattern, though with lower incidences. Investigators also reported discoloration at the application site at least once in 16% of participants, and this was the most common adverse event not captured by the eDiary. Discoloration tended to be more frequent in those with higher Fitzpatrick skin types ranging from 7% in those with type 1 or 2 to 31% in those with type 4, and 18% in those with type 5 or 6. Results were consistent across all other subgroups analyzed. Regarding reactions of potential concern, in 2 participants, vesicles at the application site were noted. One case was participant-reported, and the duration was 1 day. The second case was noted by the investigator at a study visit, and the vesicle resolved in 4 days.

**Table 1:** Treatment-Emergent Adverse Events (TEAEs) Reported in ≥2% of Participants (Safety Population, *N* = 335

SOC Preferred Term	All Adverse Events n (%) of Participants
Application site erythema^a^	316 (94.3)
Application site swelling^a^	296 (88.4)
Application site haemorrhage^a^	225 (67.2)
Application site bruise^a^	194 (57.9)
Application site pain^a^	81 (24.2)
Application site discolouration^a^	53 (15.8)
Application site pruritus^a^	52 (15.5)
Application site oedema^a^	8 (2.4)
Upper respiratory tract infection	28 (8.4)
Sinusitis	13 (3.9)
Nausea	9 (2.7)
Viral upper respiratory tract infection	7 (2.1)

A participant who experienced multiple events within a SOC was counted only once for that SOC

Adverse events were coded with MedDRA v 20.0

*SOC* System Organ Class

^a^Adverse Event was considered possibly or probably drug-related

More than 95% of application site reactions were rated as mild; none were rated as severe. More than 80% of swelling and redness incidents were rated as zero by 48 h post-application. No skin observations warranted referral to a dermatologist. For participants who treated at least 25 attacks (*n* = 96) over the course of the study, the frequency or severity of site reactions did not change appreciably from the 1st to the 5th to the 15th or to the 25th attack.

Table 2 shows AEs that led to withdrawal from the study. All events occurred in the first 6 months of the study, and 4 participants withdrew due to application site reactions. Of the 7 total reactions, 6 were mild and all lasted less than 1 day.

**Table 2:** Number of Participants with Treatment Emergent Adverse Events^a^ leading to Study Drug Withdrawal (Safety Population, *N* = 335)

Adverse Event Preferred Term^b^	M207 3.8 mg n (%)
Amenorrhoea^c^	1 (0.3)
Anxiety^c^	2 (0.6)
Application site haemorrhage	1 (0.3)
Application site pain	1 (0.3)
Application site swelling	1 (0.3)
Application site pain, erythema, haemorrhage, and swelling	1 (0.3)
Breast cancer stage II^c^	1 (0.3)
Dysphagia	1 (0.3)
Facial Pain	1 (0.3)
Hangover	1 (0.3)
Migraine	1 (0.3)
Nausea	1 (0.3)
Paraesthesia and somnolence	1 (0.3)
Pharyngeal oedema	1 (0.3)

^a^Treatment emergent adverse events are defined as any new AE that started after first patch application

^b^Adverse events were coded with MedDRA v 20.0

^c^Not Related

Neurological adverse reactions that are often associated with triptans were reported by a small percentage of participants in this study; no event was reported in 2% or more of participants. Dizziness was reported by 1.8%. Other typical triptan-associated adverse events such as paresthesia and jaw tightness were reported in 1–1.5%. There was one serious and possibly treatment-related adverse event in the trial; one participant became pregnant and had received one dose of study drug. Although unconfirmed, she may have been pregnant at the time of her dose. She reported by telephone that a birth defect in the unborn fetus had been observed on ultrasound. She declined to come to the study center to be seen for further evaluation, and subsequent attempts to reach her were unsuccessful.

No other serious treatment-related AEs or adverse events of special interest (scarring or any site reactions requiring further evaluation or care) were reported. There were no reported infections at the application site.

### Efficacy

**Fig. 2:**
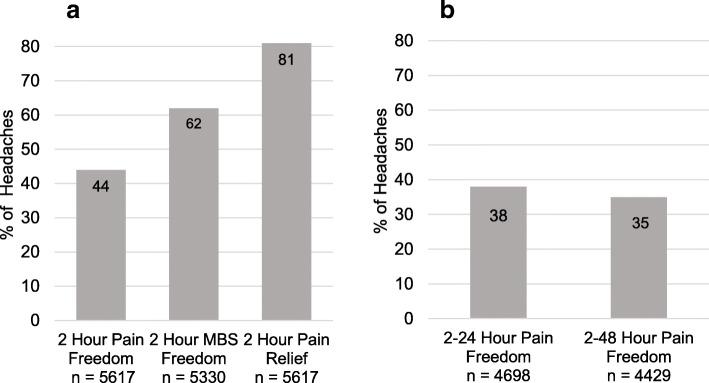
Key Efficacy Outcomes. **a** Percentage of headaches for which subjects experienced freedom from headache pain and/or most bothersome other migraine-associated symptom at 2 h post-dose. Results for MBS-freedom are presented only for those whose MBS was present pre-dose. Numbers below bars indicate number of headaches assessed. MBS: Most Bothersome Symptom. **b** Percentage of headaches for which subjects had sustained pain freedom from 2 to24 hours and from 2 to 48 h post-dose. Numbers below bars indicate number of headaches assessed

Outcomes for key efficacy variables are shown in Fig. 2. The proportion of attacks from which participants achieved pain freedom, MBS freedom, and pain relief at 2 h post-dose are very similar to what was observed in the pivotal ZOTRIP trial [2]. The proportion for which pain freedom was sustained was similar, with 38% experiencing pain freedom from 2 to 24 h and 35% from 2 to 48 h. Similarly, the percentage of attacks that had sustained pain relief 2–24 h was 70%, and for 2–48 h it was 65%. Rates for associated symptom freedom at 2 h post dose of qualifying migraine attacks were as follows: nausea 82%, photophobia 61%, and phonophobia 63%. Participants were instructed not to take any rescue medications in the first 2 h post-dose; however, rescue medication was taken within this interval in 3.6% of attacks.

## Discussion and conclusions

Safety and tolerability findings in this long-term study were similar to those seen in the previously conducted pivotal single attack ZOTRIP trial [2]. These findings are also consistent with the side effect profile that has been seen with dermatologic uses of microneedling [3, 4]. Specifically, microneedle application is associated with transient redness, swelling, and sometimes bruising, that resolves over hours to days after application. In some individuals, there can be temporary pigmentary changes that resolve typically over time. The microneedles for M207 are 340 μm in length and appear to penetrate less than 150 μm [5], which probably contributes to the transient nature of the application site effects and the lack of infections seen at the application sites. Other than application site TEAEs, the safety and tolerability were as expected with the use of zolmitriptan [6, 7].

Although application site TEAEs were generally mild, they were reported in the majority of participants in this 12-month study. Notably, investigators’ assessments tended to indicate a lower incidence; for example, the frequency of redness was 26% lower. This observation likely reflects, at least in part, the timing of the observations, as the events may have resolved by the time a participant visited the clinic. However, it should also be considered that adverse event reporting in this trial was solicited, i.e. collected via structured responses in the eDiary at 5 separate time points after patch application. This approach has the advantage of enhancing the consistency and detection of a safety signal but may also lead to inflation of reporting due to the suggestive nature of the questioning. This phenomenon has been documented in other trials in which AEs reported using a structured questionnaire were nominally similar but substantially more frequent than when participants were not asked about specific AEs [8, 9].

Photographs from preclinical studies were provided to participants to assist in assessing application site skin effects. Except for observations of concern, photography or other imaging methods were not used routinely to capture application site findings in this trial. This decision was made in accordance with discussions with the Dermatology Division at the FDA, and at least in part due to challenges associated with capturing and standardizing images.

The proportion of attacks for which pain freedom, MBS freedom, and pain relief were achieved at 2 h post-dose was similar to what was seen in the ZOTRIP trial [2]. Sustained pain freedom over the periods 2 to 24 h or 2 to 48 h was also similar (38% vs 32% for 2 to 24 h for this trial, and 35% vs 27% for 2 to 48 h for the ZOTRIP trial).

The adverse event profile of M207 is primarily related to mild skin reactions (particularly redness) that occur at the application site in a high percentage of participants. Other adverse events seen were those typically associated with zolmitriptan administration by other routes, and the rate of occurrence did not appear to be higher than with other zolmitriptan products.

Potential limitations of this study include the subjective nature of severity of participant-reported applications site reactions as well as the lack of contemporaneous assessment by investigators.

## Data Availability

De-identified individual participant data underlying the results reported in this article can be made available to researchers who provide a methodologically sound proposal, beginning 3 months and ending 5 years following article publication. Proposals should be directed to the corresponding author. To gain access, data requestors will need to sign a data access agreement.

## Abbreviations

AE: Adverse event
ICHD: International Classification of Headache Disorders
MBS: Most bothersome symptom
TEAE: Treatment-emergent adverse event
